# The Model of Mortality with Incident Cirrhosis (MoMIC) and the model of Long-term Outlook of Mortality in Cirrhosis (LOMiC)

**DOI:** 10.1371/journal.pone.0223253

**Published:** 2019-10-03

**Authors:** Ellen R Berni, Bethan I Jones, Thomas R Berni, James Whitehouse, Mark Hudson, James Orr, Pete Conway, Bharat Amlani, Craig J. Currie

**Affiliations:** 1 Global Epidemiology, Pharmatelligence, Cardiff, United Kingdom; 2 Norgine Pharmaceuticals Limited, Harefield, Uxbridge, United Kingdom; 3 Liver Unit, Freeman Hospital, Newcastle upon Tyne, United Kingdom; 4 Institute of Cellular Medicine, Newcastle University, Newcastle upon Tyne, United Kingdom; 5 Division of Population Medicine, School of Medicine, Cardiff University, United Kingdom; Medizinische Fakultat der RWTH Aachen, GERMANY

## Abstract

The purpose of this study was to produce two statistical survival models in those with cirrhosis utilising only routine parameters, including non-liver-related clinical factors that influence survival. The first model identified and utilised factors impacting short-term survival to 90-days post incident diagnosis, and a further model characterised factors that impacted survival following this acute phase. Data were from the Clinical Practice Research Datalink linked with Hospital Episode Statistics. Incident cases in patients ≥18 years were identified between 1998 and 2014. Patients that had prior history of cancer or had received liver transplants prior were excluded. Model-1 used a logistic regression model to predict mortality. Model-2 used data from those patients who survived 90 days, and used an extension of the Cox regression model, adjusting for time-dependent covariables. At 90 days, 23% of patients had died. Overall median survival was 3.7 years. Model-1: numerous predictors, prior comorbidities and decompensating events were incorporated. All comorbidities contributed to increased odds of death, with renal disease having the largest adjusted odds ratio (OR = 3.35, 95%CI 2.97–3.77). Model-2: covariables included cumulative admissions for liver disease-related events and admissions for infections. Significant covariates were renal disease (adjusted hazard ratio (HR = 2.89, 2.47–3.38)), elevated bilirubin levels (aHR = 1.38, 1.26–1.51) and low sodium levels (aHR = 2.26, 1.84–2.78). An internal validation demonstrated reliability of both models. In conclusion: two survival models that included parameters commonly recorded in routine clinical practice were generated that reliably forecast the risk of death in patients with cirrhosis: in the acute, post diagnosis phase, and following this critical, 90 day phase. This has implications for practice and helps better forecast the risk of mortality from cirrhosis using routinely recorded parameters without inputs from specialists.

## Introduction

Cirrhosis is advanced fibrosis of the liver; resulting in severe architectural distortion and derangement of liver function which can progress to portal hypertension and decompensation. Complications include ascites, variceal haemorrhage, hepatic encephalopathy, hepatorenal syndrome and hepatocellular carcinoma (HCC). The main causes of liver cirrhosis are alcohol-related liver disease (ARLD), obesity, and hepatitis B and/or C infection [1]. Morbid damage to the liver increases over time, progressing to cirrhosis over many years. Cirrhosis and HCC contribute to 2.5% of deaths worldwide, with hepatitis B the most common cause in developing countries, and ARLD being the most common cause in developed countries [2].

Cirrhosis can affect people of any age-group or gender. In the UK, mortality rates have increased four-fold since 1970, and in people under 65 years of age have risen almost five-fold [3]. Cirrhosis causes around 4,000 deaths every year in the UK [4]. Despite this, it is unclear how many people are truly affected by cirrhosis, since symptoms may arise only when the condition is clinically advanced and, often, nearly fatal [4].

Of patients who die with cirrhosis, 73% of patients present with cirrhosis who die are admitted for the first time as an emergency with decompensation [3]. The early diagnosis of cirrhosis or liver injury is therefore essential as it allows the potential for intervention which can have a significant impact on prognosis. Among patients with alcohol related cirrhosis, 65% of patients who decide to abstain from alcohol are alive at three years, as compared to virtually zero of those who continue drinking alcohol [5]. A diagnosis of compensated cirrhosis is associated with an increased risk of death 4.7 times that of the general population. The progression to decompensated liver failure is associated with a risk 9.7 times greater [6].

Elevations in liver function tests (LFTs) may identify those with liver disease but do not necessarily reflect the severity of the underlying liver disease. Alanine aminotransferase (ALT) levels may fall with progressive liver disease and may even be normal in advanced cirrhosis. The severity of liver disease is better gauged by the synthetic function, including bilirubin levels, serum albumin and prothrombin time [7]. These diagnostic tests are often documented in routine records.

In attempting to forecast survival in people with cirrhosis, there are a number of factors that are known to influence the likelihood of death in addition to the severity of the liver disease; most attributable are age and other co-morbid conditions such as cancer. A better understanding of the factors that influence the probability of death in patients with cirrhosis is needed [8].

The purpose of this study was to, produce statistical survival models at the time a patient is first diagnosed with liver cirrhosis, utilising only clinical data commonly recorded in routine clinical practice independently of any inputs from liver-disease specialists required for the existing risk models—MELD and Child-Pugh [9]. Due to the fact that many patients present at a late stage if the disease and thus there is a peak in mortality following incident diagnosis of cirrhosis. It was decided to address this differential pattern of mortality risk by developing two statistical survival models [10]. The first model was devised to identify factors associated with short-term survival to 90-days, and the second model was devised to forecast mortality-risk in patients who survived the first 90 days from diagnosis. The second model is time-dependent to ensure that patients changing from compensated to decompensated cirrhosis will be accounted for in their mortality estimate over time.

## Methods

### Data source

This study utilised general practice data from the Clinical Practice Research Datalink (CPRD) along with linked hospital episode statistics (HES) data and mortality data from the Office of National Statistics (ONS).

CPRD comprises pseudonymised data collected in a non-interventional way from participating primary-care practices throughout the UK. By January 2015, it contained records from more than 13 million research-quality patients registered at 684 practices. Data include demographics, diagnoses, symptoms, investigations, referrals and prescriptions. For more than seven million patients (54%) registered with participating English practices, their records can be linked via a trusted third party with other data sources, notably HES and the Office of National Statistics (ONS) death-certificate data.

The characteristics of patients eligible for linkage have been found to be representative of the entire data set which are, in turn, considered representative of the UK population as a whole in terms of age and gender [11,12]. This study was granted CPRD independent Scientific Advisory Committee approval (ISAC 13_114).

### Patient identification

Incident cases with cirrhosis were identified between 1998 and 2014 by either Read code in the primary care dataset or ICD-10 in the HES dataset. The diagnostic codes were validated by two clinical experts (MH and JO). These patients had to be eligible for linkage with HES and aged over 18 years at cirrhosis diagnosis (the index date). A minimum of 90 days wash-in period prior to first diagnosis of cirrhosis was applied in order to identify incident diagnosis. Patients that had prior history of cancer were excluded, and patients that received liver transplants were also excluded since this would bias the natural history of the disease.

Baseline characteristics were determined at index date. For most variables, the following algorithm was used to select their baseline characteristics: a search for the nearest value within 30 days prior to the incident cirrhotic event or, failing that, a search for the nearest value within the 30 days post index date. If a value was not found within either of these windows, the algorithm searched for the nearest reading in the 365 days prior to the index date. If more than one value was recorded on the same day, the mean was taken, with the exclusion of blood pressure readings, where the lowest systolic blood pressure (SBP) value was selected, together with its corresponding diastolic blood pressure (DBP) value.

### Morbidity prior to cirrhosis diagnosis

Since the survival models were designed to include clinical co-morbidities, and model interaction between liver disease with disease present in other major organs; prior morbid events were characterised. A history of each of the following co-morbidities was determined: cardiovascular disease (CVD), ascites, variceal haemorrhage, renal complications, serious infections (including sepsis, chest infections and urinary infections), and diabetes. Where appropriate, these clinical factors were used as binary covariables.

### Post cirrhosis events

The post diagnosis clinical events considered relevant for inclusion in the models and recorded as hospital admissions were as follows: liver disease (including hepatic encephalopathy [HE]), stroke, ascites, variceal haemorrhage, renal disease, serious infections (including sepsis, chest and urinary infections). The dataset comprised occurrence of these events post cirrhosis diagnosis on a month-by-month basis, as they were recorded. The cumulative number of admissions with these clinical events was used in the model, since a greater the number of admissions is generally considered indicative of an increasing disease severity. The only exception to this was diabetes, where a binary variable was used.

### Statistical analysis

The data were divided into two datasets. The first dataset consisted of all cirrhosis patients. The second dataset consisted of patients that survived the first 90 days. Each model was then split 80:20 in order to provide a dataset to create a training dataset, and a separate test set to determine the internal validity of the models.

Model-1 used a logistic regression model to predict death within three months. This model adjusted for prior comorbidities and decompensating events, along with other baseline characteristics and recent test results.

Model-2 used data from those patients that survived ≥3 months. Model-2 was an extension of the Cox regression model, adjusting for time-dependent covariables. This model was based on monthly, time-dependent segments, with test findings and post-index morbid events varying from month to month. All relevant model assumptions were checked. Imputation methods and model derivation details are supplied in S1 and S2 Methods.

The software used to run these analyses was R [13]. Missing data were dealt with in various ways appropriate to the parameter: exclusion of patients with missing values that could introduce bias; multiple imputation and/or other methods such as last observation carried forward (LOCF). For time-dependent data, where values were missing within the first month, the baseline value was used. LOCF was then used, with the assumption that test results would remain the same until another result was recorded. Due to patients with cirrhosis likely to change from a compensated to decompensated stage very dynamically, the time-dependent model adjusted on a month by month basis for new test result confirming this change in the patient’s cirrhosis severity. In order to use data from all patients in the analysis, including those with missing data, all continuous variables were categorised, and included a category for missing data.

### Model validation

Model-1 was validated using a confusion matrix and a receiver operator characteristic (ROC) curve. A cut-off was chosen based on the greatest sum of the specificity and sensitivity in order to increase the overall accuracy of the model. Model-2 was validated by observing ROC curves at 2, 3, 5 and 10 years, calculating the area under the curve (AUC) of the training and test data using the approach of Song and Zhou [14]. Using the training set, the mortality risk score per patient per month was split into deciles. Risk scores for each individual at each month were calculated within the test data set. The proportion of patients that died within each decile at each year were calculated.

## Results

From CPRD 26,385 patients eligible for the HES linkage scheme were identified with an incident diagnosis of cirrhosis. Of these, 11,676 (96.1%) had their diagnosis identified before their end of follow up and between 1998 and 2014. After excluding any patients that had a wash-in period of less than 90 days, 11,216 patients remained. Finally, after excluding those that had prior liver cancer and those that were under the age of 18 years old, 10,953 (97.7%) patients were included in derivation of the two models.

### Baseline characteristics

Of the 10,953 cirrhotic patients, there was a higher proportion of males than females (59.9% and 40.1%, respectively), and an overall mean age of 60.0 years (SD 14.4 years) (Table 1). Body mass index (BMI) was available for 45% of the patients, with a mean of 27.4 kg/m^2^ (SD 6.5). Most variables had between 40%—70% of data available, with aspartate aminotransferase (AST)/ALT ratio having the lowest available data at 6%. Mean AST/ALT ratio was 1.88 units (SD 1.16), mean ALT was 55.19 u/L (SD 70.27), and mean AST was 89.65 u/L (SD 93.14). Alcohol status was available for 91.1% of the cohort, with 70.5% of these also having the self-reported number of units of alcohol per week recorded. The majority of patients (70.8%) were current alcohol users, with the mean reported consumption of 24.9 alcohol units per week (SD 43.6). The most common complication among these cirrhosis patients was prior infection, with 49.3% of patients having a prior diagnosis or admission for infection. Patients also had comorbidities related to stroke (3.6%) and/or end stage renal disease (26.4%). A proportion of patients had experienced decompensating events at baseline; variceal haemorrhage (7.1%) and/or ascites (26.3%). Of the 10,953 patients, 19.5% were diabetic. The mean albumin level was 35.3 g/L (SD 7.1) and the mean bilirubin level was 39.4 μmol/L (SD 64.3). Baseline characteristics are detailed in Table 1.

**Table 1:** Baseline characteristics.

Parameter	n (%) or mean (SD)		% of missing data
Male	6,557	(60)	0
Age	60.0	(14.4)	0
Body Mass Index (kg/m^2^)	27.4	(6.5)	6027 (55.0%)
Systolic blood pressure (mm/Hg)	131	(20)	3411 (31.1%)
Total cholesterol (mmol/L)	4.6	(1.6)	6701 (61.2%)
Albumin (g/L)	35.3	(7.1)	3687 (25.3%)
Bilirubin (μmol/L)	39.4	(64.3)	3576 (32.6%)
eGFR (ml/min/1.73 m^2^)	84.9	(26.2)	3506 (32.0%)
Alanine aminotransferase (U/L)	55.2	(70.3)	4744 (43.3%)
Aspartate aminotransferase (U/L)	89.7	(93.1)	8878 (81%)
Sodium (mmol/L)	137.5	(4.6)	3570 (32.6%)
GP contacts preceding year (n)	11.5	(10.5)	0
Charlson Index	3.7	(2.4)	0
Alcohol Status:			
Never drank	1,332	(12.2)	
Ex-drinker	892	(8.1)	
Current drinker	7,758	(70.8)	
Missing	971	(8.9)	
Units of alcohol per week	24.9	(43.6)	3228 (29.5%)
Comorbidities:			
Diabetes	2,132	(19.5)	NA
Stroke	392	(3.6)	NA
End stage renal disease	2,892	(26.4)	NA
Admission for variceal haemorrhage	775	(7.1)	NA
Admission for ascites	2,885	(26.3)	NA
Admission for infection	5,396	(49.3)	NA

### Overall pattern of survival

**Fig 1:**
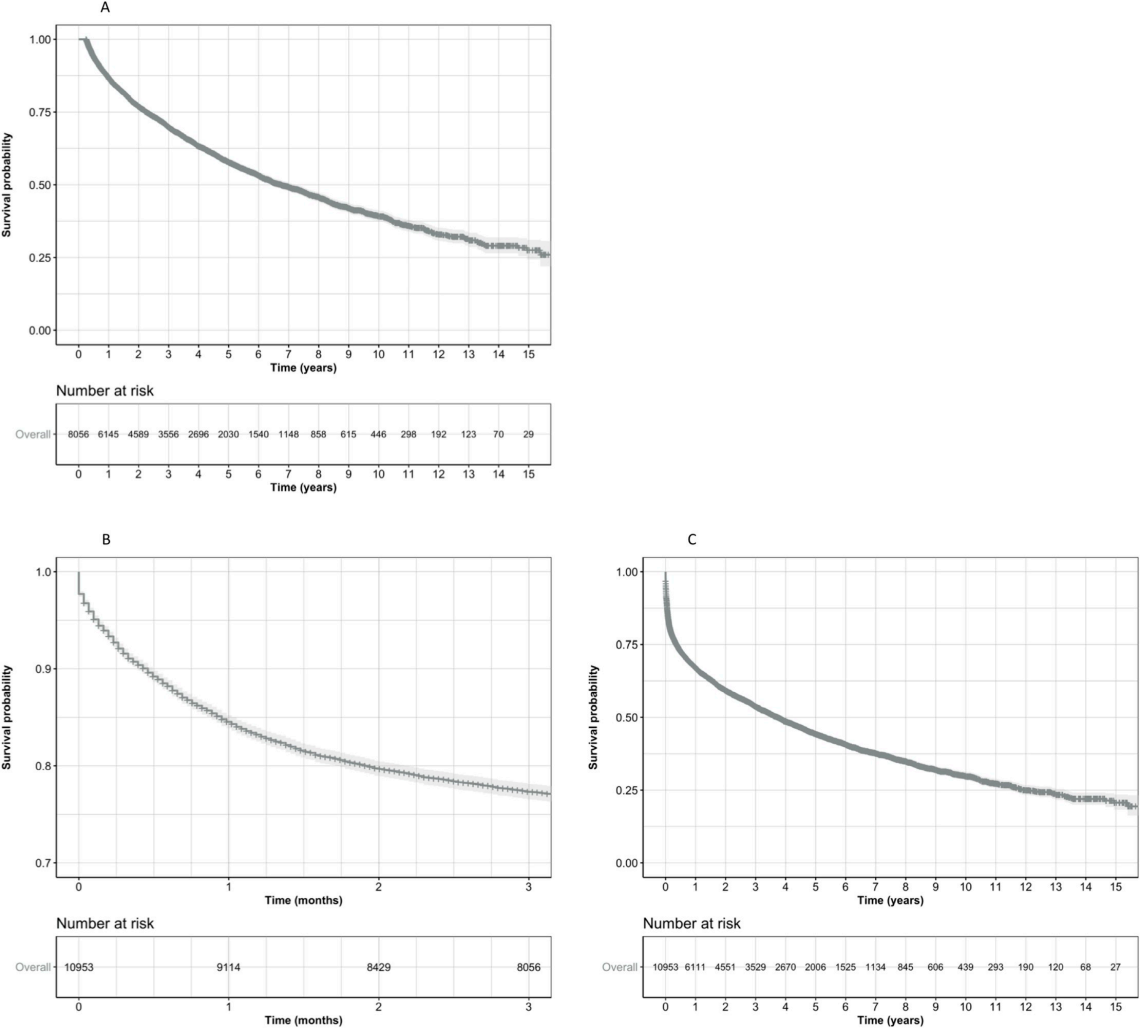
Kaplan-Meier curves illustrating survival pattern in people newly diagnosed with liver cirrhosis. (A) All subjects. (B) Survival to 90 days post diagnosis. (C) Survival in those who survived ≥ 90 days.

The postulated pattern of an increased likelihood of death in the acute phase, immediately post incident diagnosis of liver cirrhosis is evident in the Kaplan-Meier figure of overall survival in these subjects (Fig 1A). Fig 1B shows survival for the first 90 days following index date and Fig 1C shows survival from 90 days onward; illustrating why it was reasonable to adopt an approach that used two different patterns of mortality.

Overall median survival was 3.7 years. The following proportions of people were alive at 90 days, 1-year, and three-years: 77%, 68%, and 54%, respectively.

### Model-1—The Model of Mortality with Incident Cirrhosis (MoMIC): Death within 90 days post incident diagnosis

Of the selected subjects, 23% died in the immediate 90 days post diagnosis of cirrhosis (Fig 1B). Initially, all potential baseline variables were included in the model; the recommended range for each variable was used as the reference case. Prior disease history and decompensating events such as prior ascites, admissions for infections and end stage renal disease were also included in the model.

**Fig 2:**
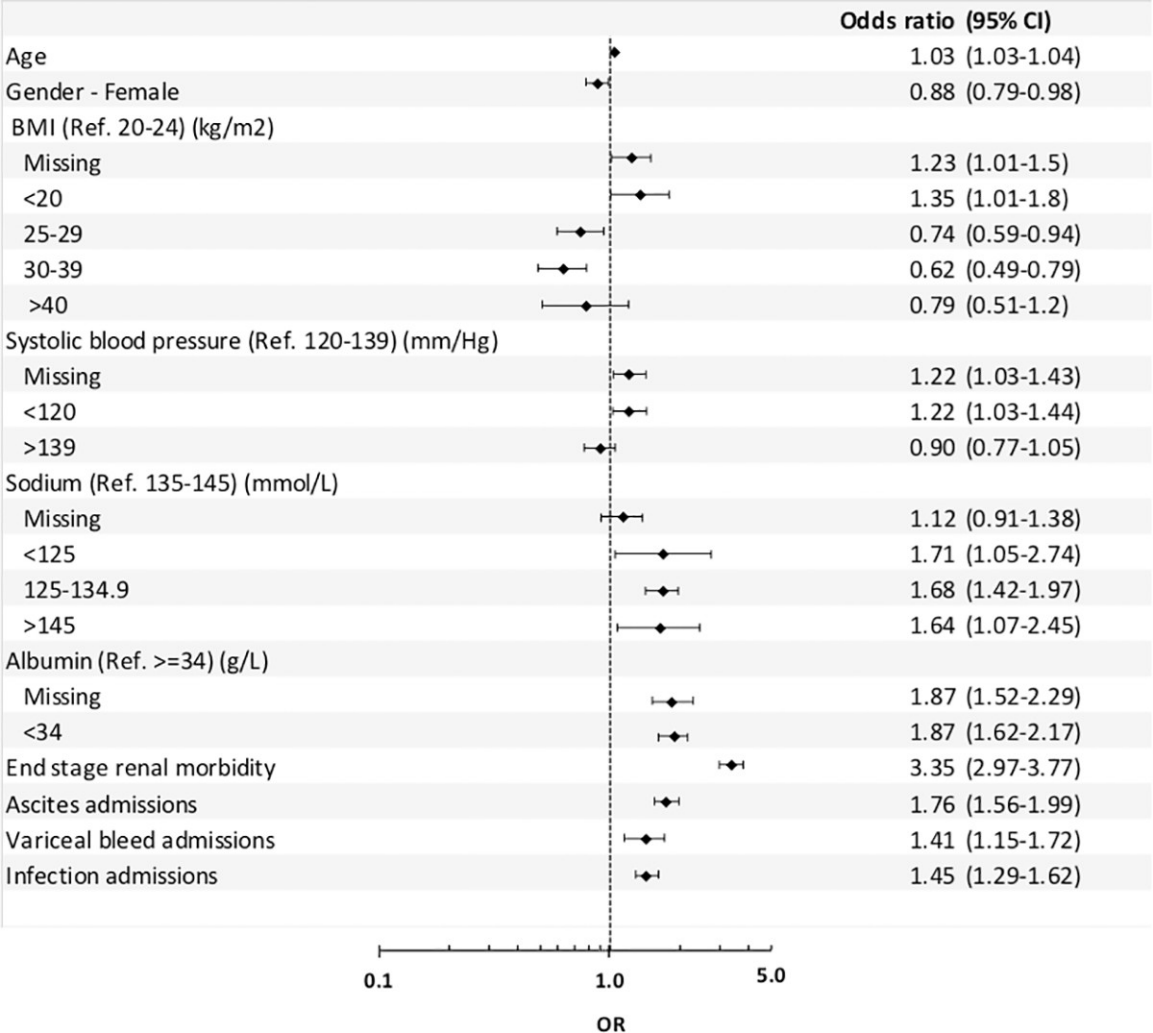
MoMIC: Logistic regression model of mortality within 90 days following incident cirrhosis.

For each year of age, the adjusted odds of death were increased by 3% (adjusted odds ratio (OR) = 1.03, 1.03–1.04) for patients diagnosed with cirrhosis (Fig 2). Being female (vs. male) with cirrhosis decreased the odds of death by 12% (OR = 0.88, 0.79–0.98, Fig 2). The reference case for body mass index (BMI) was patients with a BMI value between 20–24 kg/m^2^. Patients with a BMI of less than 20 had a 35% increased odds ratio of death in comparison to the reference case (OR = 1.35, 1.01–1.80). For all other categories of BMI—all greater than the reference case, therefore overweight—the odds ratio of death was lower than the reference group; however, the OR in those > 40kg/m^2^ was not statistically significant (Fig 2). Patients with an SBP of <120 mmHg had a 22% increased odds of death (OR = 1.22, 1.03–1.44) compared to the reference group (120–139 mmHg, Fig 2). The reference group for sodium was 135—145mmol/L. All categories of sodium with a concentration below the reference group had increased odds of mortality. Those with <125mmol/L had the highest adjusted odds ratio of 1.71 (1.05–2.74). Patients with a sodium measurement of between 125–134.9 mmol/L had an odds ratio of 1.68 (1.42–1.97, Fig 2). The reference case for albumin was greater than or equal to 34 g/L, with only one other category of less than 34 g/L. Patients with albumin measurements of less than 34 g/L had increased odds of 87% (OR = 1.87, 1.62–2.17) in comparison to those with value ≥34 g/L, this finding was highly significant (Fig 2).

A number of comorbidities and decompensating events were adjusted-for in the model, each of these resulted in a significantly increased, adjusted odds ratios for those with the comorbidities present. Patients with history of renal problems had an odds ratio of 3.35 (2.97–3.77); those with history of ascites had odds ratio of 1.76 (1.56–1.99); those with a variceal bleed history had an odds ratio of 1.41 (1.15–1.72); and those with history of infections related to liver disease had an odds ratio of 1.45 (1.29–1.62, Fig 2).

### Model-2—Long-term Outlook of Mortality in Cirrhosis (LOMiC): Estimation of the likelihood of survival at 90 days following the incident diagnosis of cirrhosis

**Fig 3:**
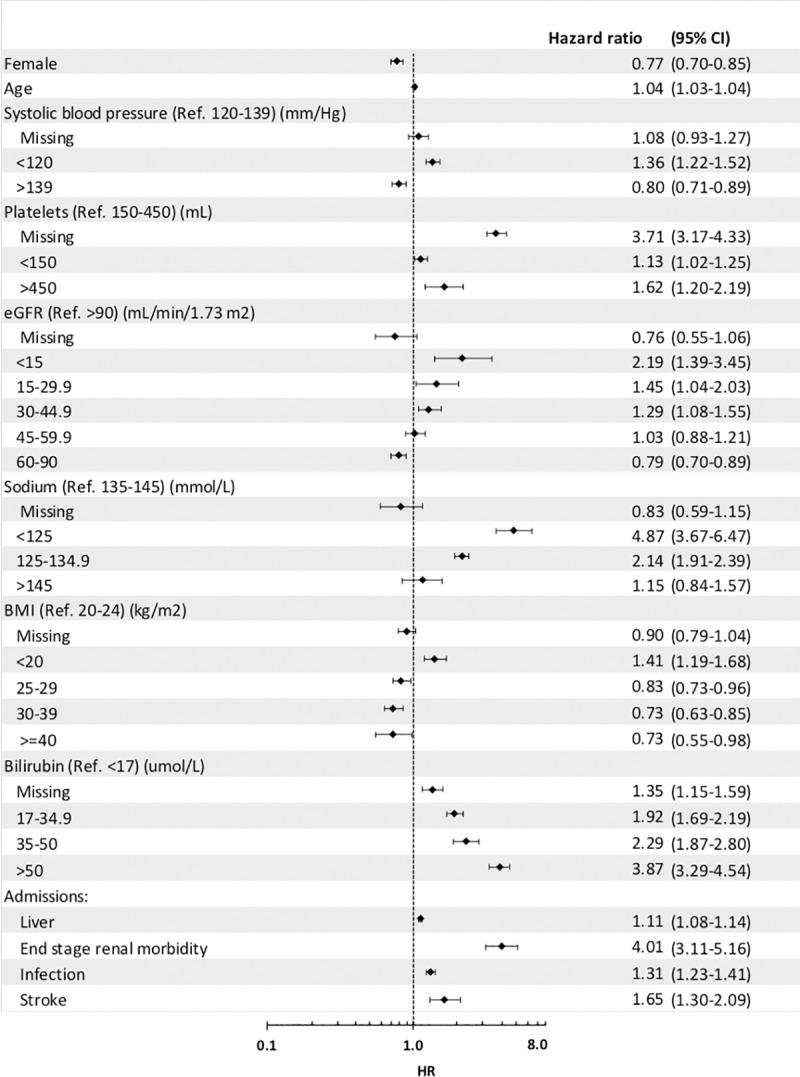
LOMiC: A time dependent Cox proportional hazard model of long-term mortality of subjects who survive ≥90 days.

Model-2, a time-dependent, Cox-proportional hazard model is detailed in Fig 3. For each year of age there was a 4% (adjusted hazard ratio (HR) = 1.04, 1.03–1.04) increased risk of death. Being female decreased the adjusted hazard of death by 23% (HR = 0.77, 0.70–0.85). Those with an SBP below the referent range (120–139 mmHg) had an increased risk of death by 36% (HR = 1.36, 1.22–1.52); those with values greater than the reference group had reduced risk of death by 20% (HR = 0.80, 0.71–0.89). The referent range for platelets was a normal platelet count of 150-450/mL. Patients with a low platelet count of <150/mL had a 13% increased risk of death when compared to the normal range (HR = 1.13 (1.02–1.25). Patients with a high platelet count of >450/mL had a significant 62% increase in the risk of death (HR = 1.62, 1.20–2.19). Patients with an eGFR value of greater than 90 ml/min/1.73 m^2^ were used as the reference group. An inverse relationship between eGFR and the hazard ratio for death was observed with a significant increase hazard ratio for eGFR groups <15 ml/min/1.73 m^2^, 15–29.9 ml/min/1.73 m^2^ and 30–44.9 ml/min/1.73 m^2^ with hazard ratios of 2.19 (1.39–3.45), 1.45 (1.04–2.03) and 1.29 (1.08–1.55), respectively. For the eGFR group 45–59.9 ml/min/1.73 m^2^ there was a 3% increase in risk of death however this result was not significant (HR = 1.03, 0.88–1.21). For eGFR group 60–90 ml/min/1.73 m^2^ there was a significant decrease in the hazard ratios with 0.79 (0.70–0.89). The reference group for sodium was between 135–145 mmol/L, with all other categories having an increased risk of death, however, >145mmol/L was not significant. Patients with a sodium measure less than 125 and 125–134.9 mmol/L had an increased risk of death with a hazard ratios of 4.87 (3.67–6.47) and 2.14 (1.91–2.39), respectively. BMI had a reference range of between 20 and 24 kg/m^2^. Patients with a BMI result <20 kg/m^2^ was the only BMI group to have an increased risk of death with a HR of 1.41 (1.19–1.68). All other BMI groups had an decreased risk of death of 17% for 25–29 kg/m^2^ (HR = 0.83, 0.73–0.96), and 27% for both 30–39 kg/m^2^ (HR = 0.73, 0.63–0.85) and ≥40 kg/m^2^ (HR = 0.73, 0.55–0.98). Patients with a measurement of bilirubin above the reference range (<17 μmol/L) had an increased risk of death. Those with the measurement 17–34.9 μmol/L, 35–50 μmol/L and greater than 50 μmol/L had a hazard ratio of 1.92 (1.69–2.19), 2.29 (1.87–2.80) and 3.87 (3.29–4.54), respectively. For each liver-related admission (including liver, ascites and variceal haemorrhage) there was a 11% (HR = 1.11, 1.08–1.14) increase in the risk of death. For each infection-related admission there was a 31% (HR = 1.31, 1.23–1.41) increase in the risk of death. For each stroke-related admission there was a 65% (HR = 1.65, 1.30–2.09) increase risk of death. Those with end stage renal disease had a 301% (HR = 4.01, 3.11–5.16) increased risk of death compared to those without.

### Model validation

Based on the greatest sum of the specificity and sensitivity (sensitivity = 0.71 and specificity = 0.67) a cut-off value of 0.23 was used for Model-1, this is consistent with previous studies [15]. Model-1 had an AUC of 0.70 (95%CI 0.69–0.71), and the ROC curve correctly predicted deaths. In order to compare the accuracy of our model to existing models we calculated the diagnostic accuracy of MELD. When calculating the MELD score on our test set, 87% of patients could not have a score calculated due to missing data. Of the 13% of patients where MELD was able to be calculated we produced a diagnostic accuracy of AUC 0.63 (95% CI: 0.55–0.70). When applying the MoMIC model on the same cohort of patients the diagnostic accuracy was 0.69 (0.62–0.76) and 0.7(0.69–0.71) on the overall test set.

**Fig 4:**
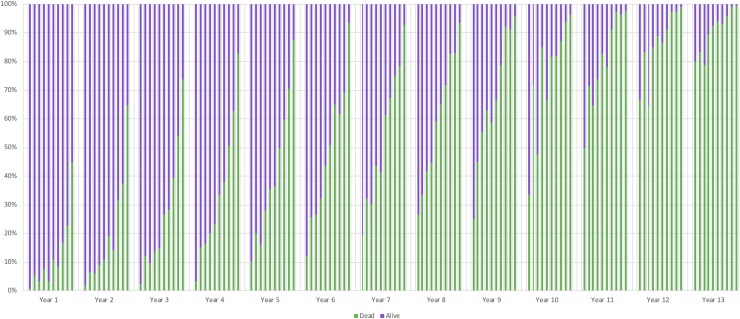
Proportion of patients dead and alive within deciles of risk score at each year in Model-2.

Model-2 showed a linear increase in both yearly deciles, and for each decile over the increasing yearly time period (Fig 4). The mortality risk score per patient per month was split into deciles and correctly predicted the pattern of survival. Within each year there was a linear increase through the decile range, with year one having a risk score of death in the first decile of 0.8%, year 3 of 2.3% and year 6 of 12.1%. There was also an increase in each decile over the years, with year 1 having 0.8% for decile one and 45.0% for the tenth decile compared to year 9, having 25.0% and 95.7%, respectively. The deciles of risk scores over the time-period indicated that the model correctly predicted deaths up to 9 years, following that the deciles converged due to the progressive nature of the disease.

## Discussion

This study derived two statistical survival models requiring input from routine healthcare data only, that forecast mortality for patients diagnosed with cirrhosis. The two-model approach was required because a large proportion of patients died within the first few months of their incident diagnosis. The two distinct statistical models forecast the likelihood of death during and after the initial phase, post-incident-diagnosis. Validation was performed on MoMIC (Model-1) and LOMiC (Model-2) separately, and both models were found to be reliable. MoMIC was correctly predicted deaths with an accuracy of 70%, whilst LOMiC correctly predicted survival up to 9 years post diagnosis. These models differ from previously published models [9] in that they adjust for progression of a patient’s morbidity, require only routine data, and are able to model separately those patients who die within 90 days of presentation.

The first 90 days of the disease was confirmed as an acutely critical phase for patients diagnosed with cirrhosis, where 23% of patients died. Over this phase, numerous factors, prior comorbidities and decompensating events were associated with mortality. All of the comorbidities that were evaluated were independently associated with increased odds of death, with prior end stage renal morbidity having the largest adjusted odds ratio (3.4). In LOMiC, cumulative hospital admissions for liver disease related diagnoses and severe infections were significantly associated with increased mortality. Renal disease was included as a binary variable, and again it was highly significant (HR = 4.01). Age, gender and albumin were significant within both MoMIC and LOMiC. this is a recognised consequence of progressive liver failure. SBP falls in these patients as a result of systemic vasodilatation, which is part of the proposed pathogenic mechanism of hepatorenal failure (HRS) [16]. This is reflected in Model-1, where lower SBP increased the odds of death (SBP <120, OR = 1.22 [1.03–1.43]). In MoMIC, the most influential variable was renal disease. Renal dysfunction is well recognised as a very important prognostic factor in cirrhosis as exemplified in the Model for End-stage Liver Disease (MELD), MELD sodium and UKELD [17]. In LOMiC, the most influential predictors were sodium less than 125 mmol/L, Bilirubin >50 μmol/L, eGFR <15 ml/min/1.73 m^2^ and prior end stage renal disease. This is in line with the UKELD score in which the serum sodium had the greatest predictive effect on outcome, this led to the creation of the MELD sodium score [18]. Other variables that increased the risk of death in LOMiC were older age, being male, low SBP, platelets >450/mL and BMI <20kg/m^2^, bilirubin levels greater than 17 μmol/L, liver disease-related admissions, and admissions for severe infections and stroke. Whilst it is widely accepted that a high platelet count is associated with increased risk of death [19], thrombocytopenia is associated with portal hypertension therefore also implying a greater risk of liver related mortality [20].

Despite there being other alternative statistical models available that also forecast the risk of death and determine a patient’s suitability for transplant (MELD and Child-Pugh scores), there are some differences when comparing our model to the Child-Pugh score. The Child-Pugh score estimates life expectancy from the severity of cirrhosis whilst considering albumin and bilirubin levels, prothrombin time, ascites and encephalopathy. Once the scores have been calculated, they are grouped into three categories that correspond to predicted life expectancy. However, due to subjective variables being used, and certain measurements not standardised, this method is considered to have certain limitations [21]. A direct comparison to Child-Pugh cannot be made due to the absence of an ICD-10 code for hepatic encephalopathy. Degré et al reported the diagnostic accuracy of 0.73 for the Child-Pugh and 0.70 for MELD [22]. The MoMIC diagnostic accuracy was 0.70.

We were able to make a direct comparison between the MELD score and the MoMIC on a subset of our test set. We found a higher diagnostic accuracy with the MoMIC than the MELD.

The MoMIC was also comparable to MELD-NA, which is a version of the MELD score that also incorporates serum sodium levels. Kartoun et al found that MELD produced an AUC of 0.69 and MELD-NA 0.70 based on 4,781 cirrhosis patients admitted to hospital and assessed patient’s mortality risk within 90 days of discharge [23].

Due to the fact that patients with cirrhosis dynamically change from compensated to decompensated states during their disease journey our LOMiC model is a unique way of re-examining a patient’s survival estimates throughout different stages of their disease. This differs from other models who only predict survival based on their baseline measurements and do not adapt for how the patients state can alter over time.

CPRD contains data collected from routine practice and, as a result, some data may be missing, and coding inaccuracies may lead to the misclassification of cirrhosis. However, within this study only patients that met CPRD’s research-quality criteria were included. Retrospective observational study designs can only be used to determine possible associations between observed events and outcomes. However, retrospective data allows large numbers of patients to be observed over a potentially long period of time without having to wait for data to be collected. Alcohol consumption status was available for 90% of the cohort; however, only 49% of these also had a corresponding value for the number of units of alcohol per week. HES-linked data was used in this study to improve case ascertainment and to follow these critically ill patients through secondary care. However, HES linked data was only available for patients registered at English practices which reduces the number of patients available for analysis. Due to the model requiring numerous parameters it may seem too complicated for day to day clinical use. However, most of the parameters required for the model should be collected as part of a patient’s routine clinical visit or can be answered from patient’s prior history. 6Where parameters are not available missing values can be entered to ensure the model can still produce a survival estimate, however this may decrease the accuracy of the result. This model will enable patients to get updated survival estimates based on their disease progression.

In conclusion, two survival models that included parameters commonly recorded in routine clinical practice were generated that reliably forecast the risk of death in patients with cirrhosis in both their acute phase post-incident diagnosis phase, and following this critical 90 day phase. This has implications for practice and may help better identify patients at risk of mortality from cirrhosis. The models aim to risk-stratify patients with cirrhosis based on other relevant diagnoses, but also have the potential to be used in economic modelling studies in the future with the aim of reducing costs.

## Supporting information

S1 Methods Imputation method. (DOCX)

S2 Methods Model derivation. (DOCX)
