# Supplementary material for: The Model of Mortality with Incident Cirrhosis (MoMIC) and the model of Long-term Outlook of Mortality in Cirrhosis (LOMiC)
Source: PLoS One. 2019 Oct 3;14(10):e0223253. doi: 10.1371/journal.pone.0223253 (PMC6776387; doi:10.1371/journal.pone.0223253)
Supplement: S1 Methods — (DOCX) [file pone.0223253.s001.docx]

**S1 Methods. Imputation method**

Since LOMiC is a monthly updated time-dependent model there is a need to impute missing values for months in which no measurements are recorded. Last observation carried forward (LOCF), by which the measurement recorded in the prior time window is carried forward until another measurement is recorded, was used to impute all missing values.
