# Supplementary material for: The Model of Mortality with Incident Cirrhosis (MoMIC) and the model of Long-term Outlook of Mortality in Cirrhosis (LOMiC)
Source: PLoS One. 2019 Oct 3;14(10):e0223253. doi: 10.1371/journal.pone.0223253 (PMC6776387; doi:10.1371/journal.pone.0223253)
Supplement: S2 Methods — (DOCX) [file pone.0223253.s002.docx]

**S2 Methods. Model derivation**

Model derivation methods were the same in both MoMIC and LOMiC. A list of possible variables was decided upon ‘a priori’ with the advice from clinical experts in the field. These variables were selected and categorised into a suitable format as per medical literature. All ’a priori’ variables were initially included in the model, and a stepwise backwards ANOVA method was used to determine which variable should be excluded from the model. This model was then checked for significant variables, and only significant variables were included in the final model. Variables excluded from the MoMIC model were number of GP contacts in the year prior, platelet count, prior CVD, prior diabetes, bilirubin, gamma-glutamyl transferase, alcohol status and ALT. Variables excluded from LOMiC were ALT, gamma-glutamyl transferase, total cholesterol, AST, alcohol status and prior diabetes.
